# Transfer of *Mycoplasma hyopneumoniae*-specific cell mediated immunity to neonatal piglets

**DOI:** 10.1186/s13567-021-00968-0

**Published:** 2021-06-30

**Authors:** Evelien Biebaut, Lisa Beuckelaere, Filip Boyen, Freddy Haesebrouck, Charles-Oliver Gomez-Duran, Bert Devriendt, Dominiek Maes

**Affiliations:** 1 Department of Reproduction, Obstetrics and Herd Health, Faculty of Veterinary Medicine, Ghent University, Salisburylaan 133, 9820 Merelbeke, Belgium; 2 Department of Pathology, Bacteriology and Avian Diseases, Faculty of Veterinary Medicine, Ghent University, Salisburylaan 133, 9820 Merelbeke, Belgium; 3 Department of Virology, Parasitology and Immunology, Faculty of Veterinary Medicine, Ghent University, Salisburylaan 133, 9820 Merelbeke, Belgium; 4 Boehringer Ingelheim Vetmedica GmbH, Binger Strasse 173, 55216 Ingelheim, Germany

**Keywords:** *Mycoplasma hyopneumoniae*, maternal immunity, cell mediated immunity, cross-fostering

## Abstract

*Mycoplasma hyopneumoniae* is the primary agent of enzootic pneumonia in pigs. Although cell mediated immunity (CMI) may play a role in protection against *M. hyopneumoniae*, its transfer from sows to their offspring is poorly characterized. Therefore, maternally-derived CMI was studied in piglets from vaccinated and non-vaccinated sows. The potential influence of cross-fostering before colostrum ingestion on the transfer of CMI from dam to piglets was also investigated. Six *M. hyopneumoniae* vaccinated sows from an endemically infected herd and 47 of their piglets, of which 24 piglets were cross-fostered, were included, as well as three non-vaccinated control sows from an *M. hyopneumoniae*-free herd and 24 of their piglets. Vaccinated sows received a commercial bacterin intramuscularly at 6 and 3 weeks prior to farrowing. The TNF-α, IFN-γ and IL-17A production by different T-cell subsets in blood of sows, colostrum and blood of piglets was assessed using a recall assay. In blood of sows cytokine producing T-cells were increased upon *M. hyopneumoniae* vaccination. Similarly, *M. hyopneumoniae*-specific T-cells were detected in blood of 2-day-old piglets born from these vaccinated sows. In contrast, no *M. hyopneumoniae*-specific cytokine producing T-cells were found in blood of piglets from control sows. No difference was found in *M. hyopneumoniae*-specific CMI between cross-fostered and non-cross-fostered piglets. In conclusion, different *M. hyopneumoniae*-specific T-cell subsets are transferred from the sow to the offspring. Further studies are required to investigate the role of these transferred cells on immune responses in piglets and their potential protective effect against *M. hyopneumoniae* infections.

**Supplementary Information:**

The online version contains supplementary material available at 10.1186/s13567-021-00968-0.

## Introduction

*Mycoplasma hyopneumoniae* (*M. hyopneumoniae*) is the primary agent of enzootic pneumonia in pigs, causing significant economic losses in swine production worldwide [1, 2]. Colonization with *M. hyopneumoniae* may occur already during the first weeks of life, and dam-to-piglet transmission has been shown to be a major transmission route [3, 4]. Maternally-derived immunity (MDI) might confer some protection against *M. hyopneumoniae* colonization in piglets [5, 6]. As pigs have an epitheliochorial placenta, piglets depend on the ingestion of colostrum to receive MDI [7]. Colostral immunity consists of a humoral component and a cellular component. In addition to macrophages and neutrophils, colostrum also contains lymphocytes which can be divided in B-cells and different T-cell subsets; CD8^+^, CD4^+^, CD8^+^CD4^+^ and CD8^−^CD4^−^ [8, 9]. Antibodies and lymphocytes are transferred from sows to piglets via the colostrum [10]. Antibodies can be taken up by the piglets from colostrum originating from their mother sow or from another cross-fostered sow. However, colostral immune cells are considered to be taken up only from colostrum coming from the own mother via yet unknown mechanisms [11–13]. Besides antibodies and lymphocytes, also cytokines are transferred from sow to piglet via colostrum [14].

Vaccination of sows during gestation increases vaccine-specific antibodies in serum and colostrum, which are then transferred to the suckling piglets [6, 15, 16]. Those piglets have higher serum antibody concentrations and are less often colonized with *M. hyopneumoniae* at weaning compared to piglets from non-vaccinated sows and gilts [5, 6].

Nevertheless, cell mediated immunity (CMI) might play a more important role in the protection against *M. hyopneumoniae* as serum antibody levels are not correlated with the degree of protection [17–21]. Although a positive effect of sow vaccination on *M. hyopneumoniae* colonization in piglets has been shown, it is not commonly practiced [22].

In a previous study [23], *M. hyopneumoniae* vaccination of sows resulted in the presence of vaccine-specific lymphocytes in colostrum, which were able to proliferate after in vitro *M. hyopneumoniae* stimulation. These cells were transferred to their offspring because after colostrum intake, the lymphocytes, isolated from the blood of the piglets, proliferated as well after in vitro stimulation. When piglets from vaccinated dams were injected intradermally with purified killed *M. hyopneumoniae* (300 µg/mL in 0.1 mL) antigen, a delayed-type hypersensitivity (DTH) reaction was observed, indicating the presence of *M. hyopneumoniae* specific Th1 cells [23]. This DTH reaction was not present in piglets that were cross-fostered between *M. hyopneumoniae* vaccinated sows [13]. It is well known that vaccination of piglets against *M. hyopneumoniae* can activate T-cells [18, 20, 21, 24], whereas the influence of sow vaccination on the sow’s CMI has not been evaluated yet. Those T-cells play a central role in the host immunity against pathogens, as activated T-cells influence both innate and adaptive immunity [25]. Activated T-cells produce cytokines like tumor necrosis factor alpha (TNF-α), which is an activation marker, and Th1 cells produce interferon gamma (IFN-γ) whereas Th17 cells produce interleukin 17A (IL-17A) [26, 27]. To gain better insights into immune responses of young piglets upon *M. hyopneumoniae* vaccination and infection, it might be important to characterize the different *M. hyopneumoniae*-specific T-cell subsets and their ability to produce cytokines in blood of sows, colostrum and the transfer to neonatal piglets [6, 28, 29].

The objectives of this study were first to investigate if vaccination of sows with an inactivated *M. hyopneumoniae* vaccine may activate T-cells in the sows and second to explore if these *M. hyopneumoniae*-specific T-cells are transferred to their offspring. Therefore, *M. hyopneumoniae*-specific humoral and cell-mediated immunity in blood of sows, colostrum and blood of 2-day-old piglets were investigated. The presence of IFN-γ, TNF-α, and IL-17A producing T-cell subsets and their proliferative ability after in vitro stimulation with *M. hyopneumoniae* antigen were analyzed. The potential influence of cross-fostering before colostrum ingestion on the transfer of CMI from dam to piglets was also investigated.

## Materials and methods

### Study population and animal experiments

**Figure 1:**
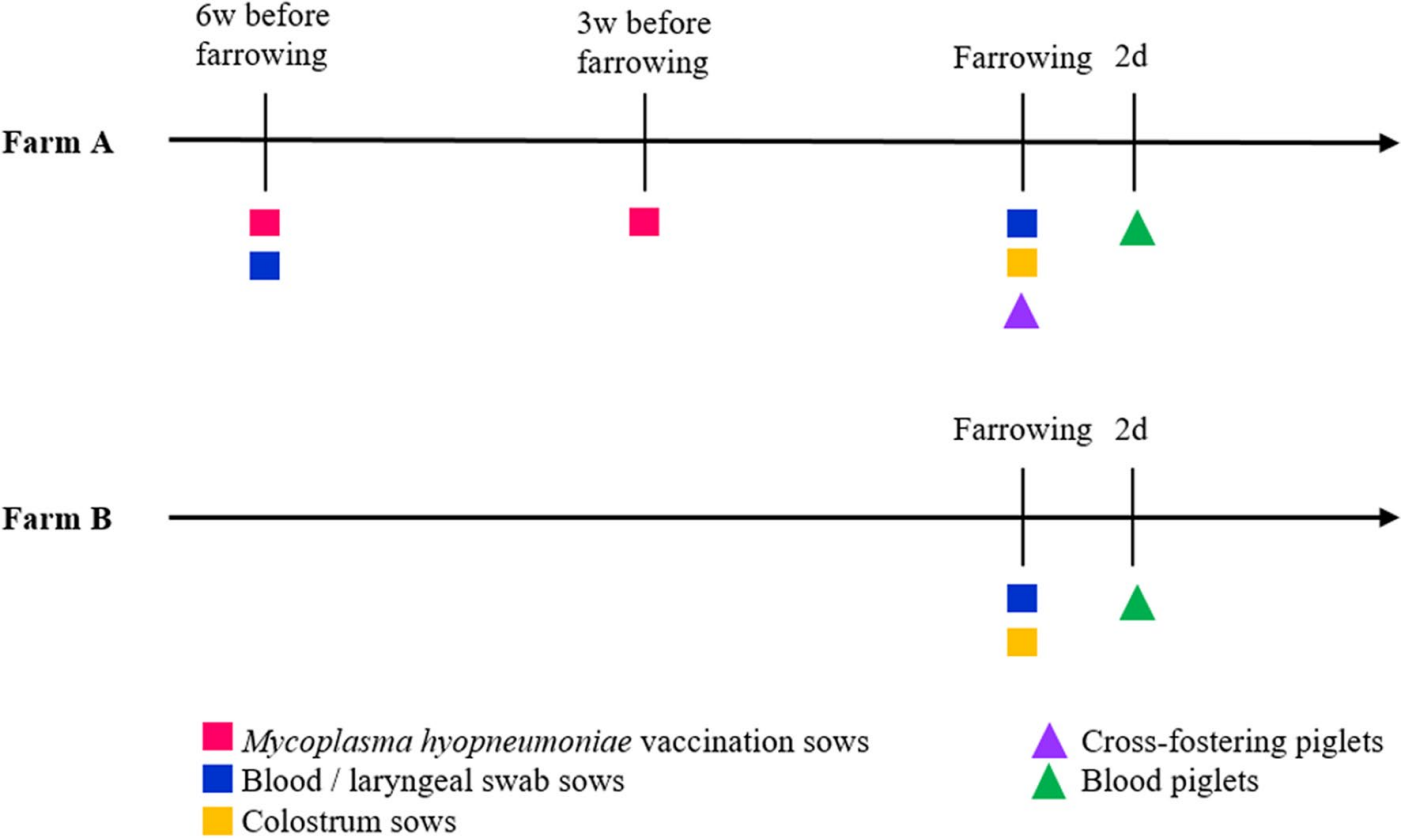
**Design of the study.** Farm A: endemically infected with *M. hyopneumoniae*; Farm B: free of *M. hyopneumoniae*. On farm A, six sows and 47 piglets were included (23 non-cross-fostered and 24 cross-fostered before colostrum ingestion), while on farm B three sows and 24 non-cross-fostered piglets were included. w = weeks, d = days.

The study was performed after approval by the Ethical Committee for Animal Experiments of the Research Institute for Agriculture, Fisheries and Food (ILVO, Merelbeke, Belgium) (approval number 2019/339) and after approval by the Ethical Committee of the Faculty of Veterinary Medicine and the Faculty of Bioscience Engineering, Ghent University (approval number 2019/12). Two commercial farrow-to-finish farms were included in the study. Farm A was endemically infected with *M. hyopneumoniae* (pathogen isolated from slaughterhouse lungs) and farm B was free of *M. hyopneumoniae.* The free status was based on historic information such as absence of clinical signs, lung lesions and serum antibodies upon routine serological testing. The farm also tested negative for *M. hyopneumoniae* using PCR on tracheobronchial swabs (pigs at 10, 14, 18 and 22 weeks of age) prior to onset of the study. The experimental design used on the two farms is shown in Figure 1.

On farm A, six sows (mixed breed and of different parities) were intramuscularly vaccinated with a commercial *M. hyopneumoniae* vaccine, constituted of inactivated whole cells of the J strain (Ingelvac MycoFLEX®, Boehringer Ingelheim Vetmedica GmbH, Ingelheim am Rhein, Germany), at 6 and 3 weeks before farrowing to boost *M. hyopneumoniae*-specific immune responses. Farrowing was induced at 115 days of gestation by intramuscular injection of 2 mL prostaglandin (Dinolytic 5 mg/mL, Zoetis, Leuven, Belgium) followed by intramuscular injection of 1 mL oxytocin 24 h later (Oxytocine Kela 10 i.u./mL, Kela, Hoogstraten, Belgium). Immediately after birth and prior to colostrum ingestion, piglets were placed in plastic tubs under a heat lamp for maximum six hours. From each sow, the first seven to eight piglets (*n* = 47) with a birth weight of at least 1.0 kg were ear notched to allow individual identification. Four piglets (*n* = 24) from each sow were moved to another dam within 6 h after birth and before colostrum ingestion, while the other three to four piglets (*n* = 23) remained with their mother.

On farm B, three Naima sows and eight piglets per sow (*n* = 24) with a birth weight of at least 1.0 kg were included in the study. No *M. hyopneumoniae* vaccination was performed on the farm and piglets were not cross-fostered.

On farm A, blood was collected in sterile serum (clotted blood) and EDTA tubes (non-clotted blood), from all sows before the first vaccination and within 6 h after farrowing. In farm B, blood was only collected from the sows within 6 h after farrowing. In addition to the blood samples, the sows on both farms were sampled using a laryngeal swab (Portex® Dog Catheter with Female Luer Mount, Smiths Medical International Ltd., Kent, UK). Colostrum (45 mL) from all sows in the study was collected shortly after birth and before the piglets were allowed to suckle. Different teats (pectoral, abdominal and inguinal) were sampled and the colostrum samples were pooled to obtain one sample per sow.

At 2 days of age, blood was taken from all piglets by puncture of the jugular vein in sterile serum and EDTA tubes.

### Nested PCR for *M. hyopneumoniae* DNA detection

To test for the presence of *M. hyopneumoniae*, DNA was extracted from the laryngeal swabs using a commercial kit (DNeasy® Blood & Tissue kit, Qiagen, Venlo, The Netherlands) and a nested PCR (nPCR) was performed [30].

### *Mycoplasma hyopneumoniae* specific antibodies

The serum was analyzed for the presence of *M. hyopneumoniae* antibodies with a commercial blocking ELISA (IDEIA™ Mycoplasma hyopneumoniae EIA kit, Oxoid Limited, Hampshire, UK) following the manufacturer’s instructions. Samples were considered positive if the optical density (OD) of the sample was lower than 50% of the average OD of the buffer control. Samples were considered negative if the OD of the sample was equal to or higher than 50% of the average OD of the buffer control. To determine *M. hyopneumoniae*-specific immunoglobulin (Ig) G and IgA levels in serum and colostrum, an indirect in-house ELISA was used as described by others [24]. Serum was diluted 1/200 for IgG and 1/100 for IgA, while colostrum was diluted 1/100 for both antibodies. All samples were tested in duplicate. Samples were considered positive if the average OD of the duplicates was higher than the cut-off value calculated as the average of the OD of the negative control samples (serum of *M. hyopneumoniae* negative pigs from experimental trials) plus three times the standard deviation (SD).

### T cell cytokine production

Peripheral blood mononuclear cells (PBMCs) were isolated from fresh, unclotted blood using a Lymphoprep™ density gradient (Stemcell technologies, Vancouver, Canada). Colostral mononuclear cells (CMCs) were purified using a Ficoll-Paque® density gradient (GE Healthcare, Illionis, USA). The stimulation and staining of the PBMCs and CMCs was based on the protocol described by others [24]. Briefly, cells were plated in 24-well plates at 2.5 × 10^6^ cells/well in 0.5 mL of AIM-V medium (Gibco™, ThermoFisher Scientific, Waltham, MA, USA) and stimulated in vitro overnight (20 h) with 3.125 × 10^7^ CCU of *M. hyopneumoniae* J strain bacterin in 0.5 mL of AIM-V medium. The *M. hyopneumoniae* J strain bacterin was made based on the protocol for the production of *M. hyopneumoniae* F7.2C bacterin [31]. Concanavalin A (10 µg/mL, Sigma-Aldrich, Saint Louis, MO, USA) stimulation was used as a positive control and AIM-V medium as a negative control. Due to low cell yields from the colostral samples and blood of 2-day-old piglets, some samples lacked enough cells to include a negative and positive control. To investigate TNF-α, IFN-γ and IL-17A production, protein secretion was inhibited by adding Brefeldin A (eBioscience, San Diego, CA, USA) to each well for the last 4 h of stimulation. Subsequently, cells were harvested and stained. First, cells were incubated with a LIVE/DEAD™ Fixable Aqua Dead Cell Stain Kit (Invitrogen™, ThermoFisher Scientific, Waltham, MA, USA) followed by incubation with anti-CD4 (clone 74-12-4) and anti-CD8α (clone 11-295-33) monoclonal antibodies. Next, corresponding secondary antibodies anti-mouse IgG2b FITC (Biolegend, San Diego, CA, USA) and anti-mouse IgG2a PE-Cy7 (Abcam, Cambridge, UK) were added together with anti-CD3 DyLight755 (clone PPT3, in house labeling). Following surface staining, fixation and permeabilization (BD Fix/Perm, Becton Dickinson, Franklin Lakes, NJ, USA), intracellular cytokine staining was performed by staining the cells with anti-human TNF-α AlexaFluor 647 (clone Mab11, Biolegend, San Diego, CA, USA), anti-pig IFN-γ PerCP-Cy5.5 (clone P2G10, BD Pharmingen™, Becton Dickinson, Franklin Lakes, NJ, USA) and anti-human IL-17A PE (clone SCPL1362, BD Pharmingen™, Becton Dickinson, Franklin Lakes, NJ, USA). Since no IL-17A production was observed upon stimulation of PBMCs from 2-day-old piglets of farm A, for piglets of farm B, IL-17A staining was omitted allowing us to stain CD3 with primary anti-CD3 (clone PPT3) and anti-mouse IgG1 PE (Biolegend, San Diego, CA, USA). Before the start of the cytokine staining, an additional blocking step was performed with mouse IgG1 (10 µg/mL). Data were acquired with a CytoFLEX flow cytometer (Beckman Coulter, Bea, CA, USA) and the results were further analyzed with CytExpert software (Beckman Coulter). The gating hierarchy is shown in Additional file 1.

### T cell proliferation assay

PBMCs were labeled using a CellTrace™ Cell Proliferation Kit (Invitrogen™, ThermoFisher Scientific, Waltham, MA, USA) following the manufacturer’s instructions. Afterwards, cells were plated in 24-well plates at 1 × 10^6^ cells/well in 0.5 mL complete cell culture medium (Dulbecco’s Modified Eagle Medium, 10% fetal calf serum, 1% penicillin/streptomycin, 1% nonessential amino acids) and stimulated in vitro for 87 h with 3.125 × 10^7^ CCU of *M. hyopneumoniae* J strain bacterin. Concanavalin A (10 µg/mL) was used as a positive control and complete cell culture medium as a negative control. Cells were harvested and a surface staining protocol was performed. First, cells were incubated with anti-CD4 and anti-CD8α monoclonal antibodies and subsequently with the corresponding secondary antibodies anti-mouse IgG2b FITC (Biolegend, San Diego, CA, USA) and anti-mouse IgG2a AlexaFluor 647 (Biolegend, San Diego, CA, USA) together with anti-CD3 DyLight755 and propidium iodide. Data were acquired with a CytoFLEX flow cytometer and the results were further analyzed with CytExpert software. The gating hierarchy is shown in Additional file 2.

### Data analyses

Statistical analyses were performed using IBM SPSS® Statistics Version 26 (IBM, Chicago, IL, USA). Kolmogorov–Smirnov and Shapiro–Wilk tests were used as tests for normality distribution of the residuals. The paired-samples T-test or the Wilcoxon signed-rank test were used for analyzing statistical differences in CMI parameters in blood of sows before and after vaccination. The independent-samples T-test or Mann–Whitney U test were used for comparing the CMI data (1) from sows and 2-day-old piglets on farm A and B and (2) from 2-day-old non-cross-fostered and cross-fostered piglets. To investigate correlations in CMI between sows and 2-day-old piglets, a Spearman-rank test was performed. Differences were considered statistically significant if the *P*-value was lower than 0.05.

## Results

### *M. hyopneumoniae*-specific antibodies

#### Commercial ELISA

To investigate *M. hyopneumoniae*-specific antibodies in the sows upon *M. hyopneumoniae* vaccination and their transfer to piglets via colostrum, antibody levels were assessed in colostrum and serum of sows and piglets. At the time of farrowing, all sows and 2-day-old piglets, were positive for *M. hyopneumoniae* specific antibodies on farm A, whereas all animals were negative on farm B (Additional file 3).

#### Isotype-specific ELISA

**Figure 2:**
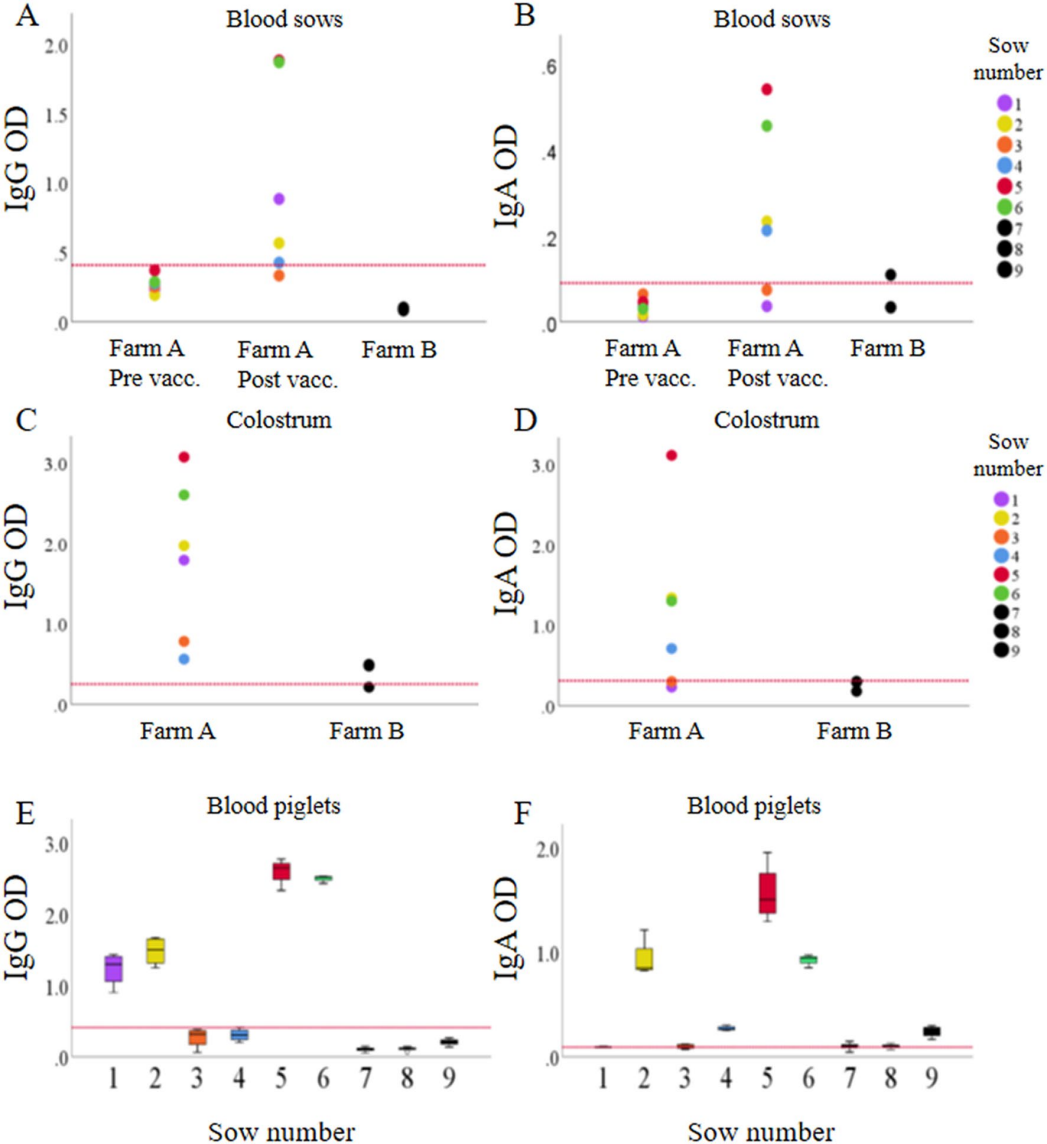
***Mycoplasma hyopneumoniae-***specific IgG and IgA levels in sows and piglets. Farm A: endemically infected with *M. hyopneumoniae*; Farm B: free of *M. hyopneumoniae*. **A**,** B**: individual levels in serum of sows; **C**,** D**: individual levels in colostrum of sows; **E**,** F**: average levels in serum of 2-day-old piglets (4 piglets per sow on farm A; 8 piglets per sow on farm B). On farm A, sows (*n* = 6) were vaccinated against *M. hyopneumoniae* at 6 and 3 weeks before farrowing and blood samples were taken before the first vaccination (pre vacc.) and at the time of farrowing (post vacc.). On farm B, blood of the sows (*n* = 3) was sampled at the time of farrowing. Color coding of the piglets corresponds to the color of their mother of which they ingested colostrum. Red line: cut-off optical density value for positive samples.

To further evaluate the vaccine boosted antibody responses, *M. hyopneumoniae*-specific serum IgG and IgA levels in sows were determined. As shown in Figures 2A and B, vaccination of sows on farm A increased both IgG and IgA levels in most animals. One sow (sow 3) remained negative for both *M. hyopneumoniae*-specific IgG and IgA, while another sow (sow 1) did not show an IgA response after vaccination. On farm B one sow had *M. hyopneumoniae*-specific serum IgA levels above the threshold (Figures 2A and B). *M. hyopneumoniae*-specific IgG and IgA levels in colostrum were detected in all sows on farm A, while the levels were below or just above the threshold for the sows on farm B (Figures 2C and D). In serum of 2-day-old piglets, the *M. hyopneumoniae*-specific antibody levels reflected the levels observed in the serum of the respective sows. Piglets with antibody levels below the threshold were born from sows with no or a low IgG or IgA response upon vaccination and low antibody levels in colostrum. Two sows (sow 3 and 4) had low IgG levels in colostrum and their piglets had serum IgG levels below the threshold. Two sows (sow 1 and 3) had low IgA levels in colostrum and their piglets had serum IgA levels below the threshold. On farm B piglets from one sow had IgA levels in serum above the threshold, this sow had serum IgA levels above and colostrum IgA levels below the threshold. An overview of the antibody concentrations in serum of 2-day-old piglets is given in Figures 2E and F. In summary, the results of the commercial ELISA and the isotype-specific ELISA in farm A showed transfer of vaccine-boosted maternal antibodies to piglets via colostrum.

### PCR testing for* M. hyopneumoniae* on laryngeal swabs

All laryngeal swabs from the sows taken 6 weeks before farrowing (farm A) and taken at farrowing (farm A and B) tested negative for the presence of *M. hyopneumoniae* DNA.

### T-cell subsets

The frequency of the different T-cell subsets after in vitro *M. hyopneumoniae* stimulation was investigated.

**Figure 3:**
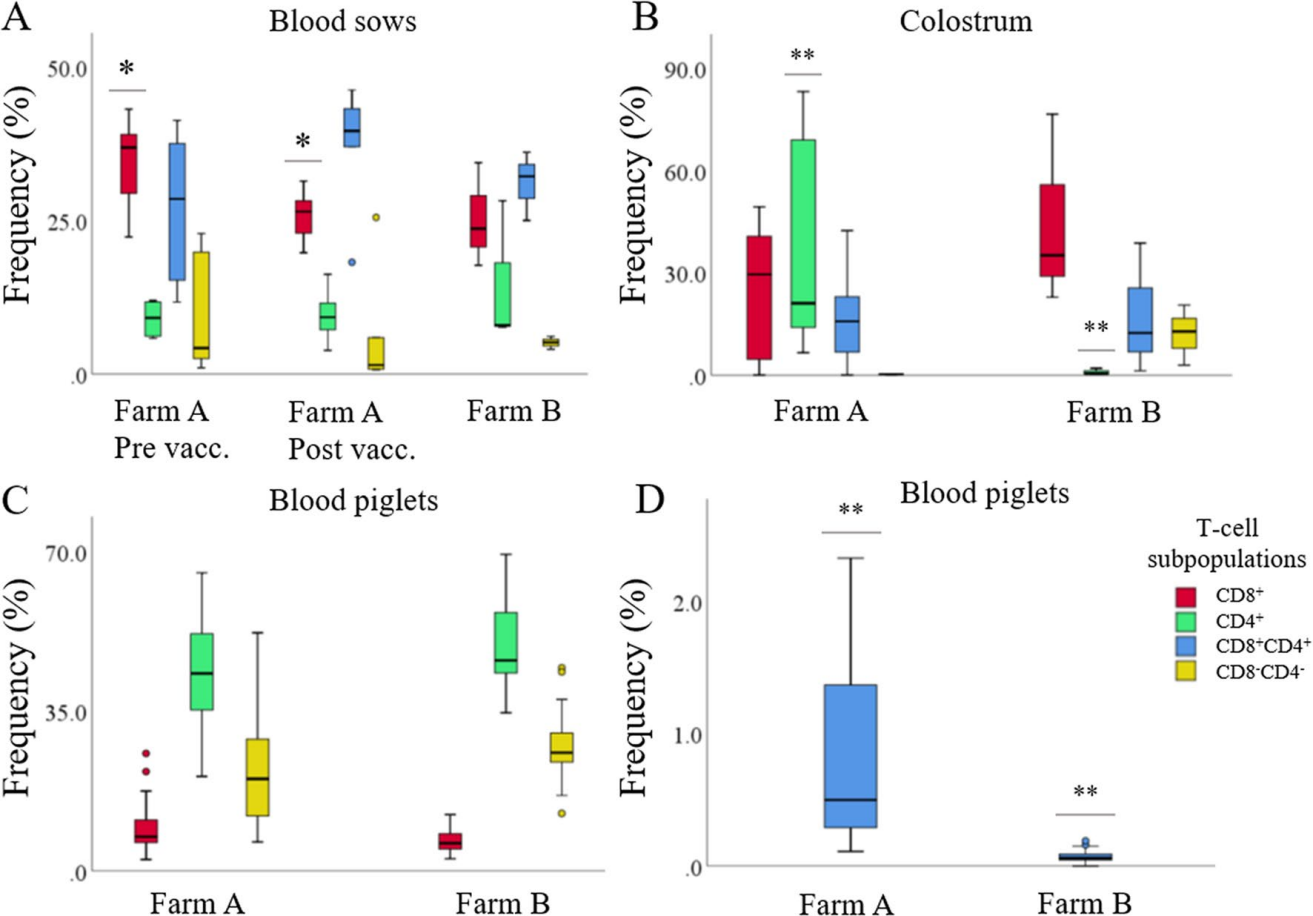
**Frequencies (%) of CD3**^**+**^** T-cells in sows and piglets.** Farm A: endemically infected *M. hyopneumoniae;* Farm B: free of *M. hyopneumoniae*. **A** Blood of sows; **B** colostrum; **C**, **D** blood of 2-day-old piglets (4 piglets per sow on farm A; 8 piglets per sow on farm B). On farm A, sows (*n* = 6) were vaccinated against *M. hyopneumoniae* at 6 and 3 weeks before farrowing and blood samples were taken before the first vaccination (pre vacc.) and at the time of farrowing (post vacc.), on farm B blood was sampled of the sows (*n* = 3) at the time of farrowing. *, *P* < 0.05 between pre- and post-vaccination on farm A; **, *P* < 0.05 between farm A and B.

*M. hyopneumoniae* vaccination induced a significant (*P* = 0.03) decrease in the frequency (average % ± SD) of peripheral blood CD8^+^ T-cells in sows (Figure 3A). The frequency of CD8^+^ T-cells in blood of sows after in vitro stimulation was 34.6% ± 7.6 and 25.9% ± 4.1 before vaccination and 6 weeks after vaccination, respectively. At the time of farrowing, the dominant T-cell population in blood of sows was CD8^+^CD4^+^ cells on both farms, while CD8^−^CD4^−^ T-cells had the lowest frequency. No significant differences were observed in the frequencies of the different T-cell populations in blood of sows on farm A and B at the time of farrowing (Figure 3A).

In colostrum of sows on farm A, CD4^+^ T-cells were dominant, whereas on farm B almost no CD4^+^ T-cells were present, this difference was significant (*P* = 0.04) (Figure 3B).

In blood of 2-day-old piglets from both farms, the dominant T-cell populations were CD4^+^ T-cells, followed by CD8^−^CD4^−^ T-cells, CD8^+^ T-cells, and CD8^+^CD4^+^ T-cells. The only significant difference between piglets on both farms was observed for the CD8^+^CD4^+^ T-cells (*P* < 0.01) with a frequency of 0.9% ± 0.7 and 0.07% ± 0.05, on farm A and B, respectively (Figures 3C and D).

### T-cell cytokine production

To investigate the transfer of CMI from mother to offspring, the cytokine production by different T-cell subsets of PBMCs was assessed in recall assays.

**Figure 4:**
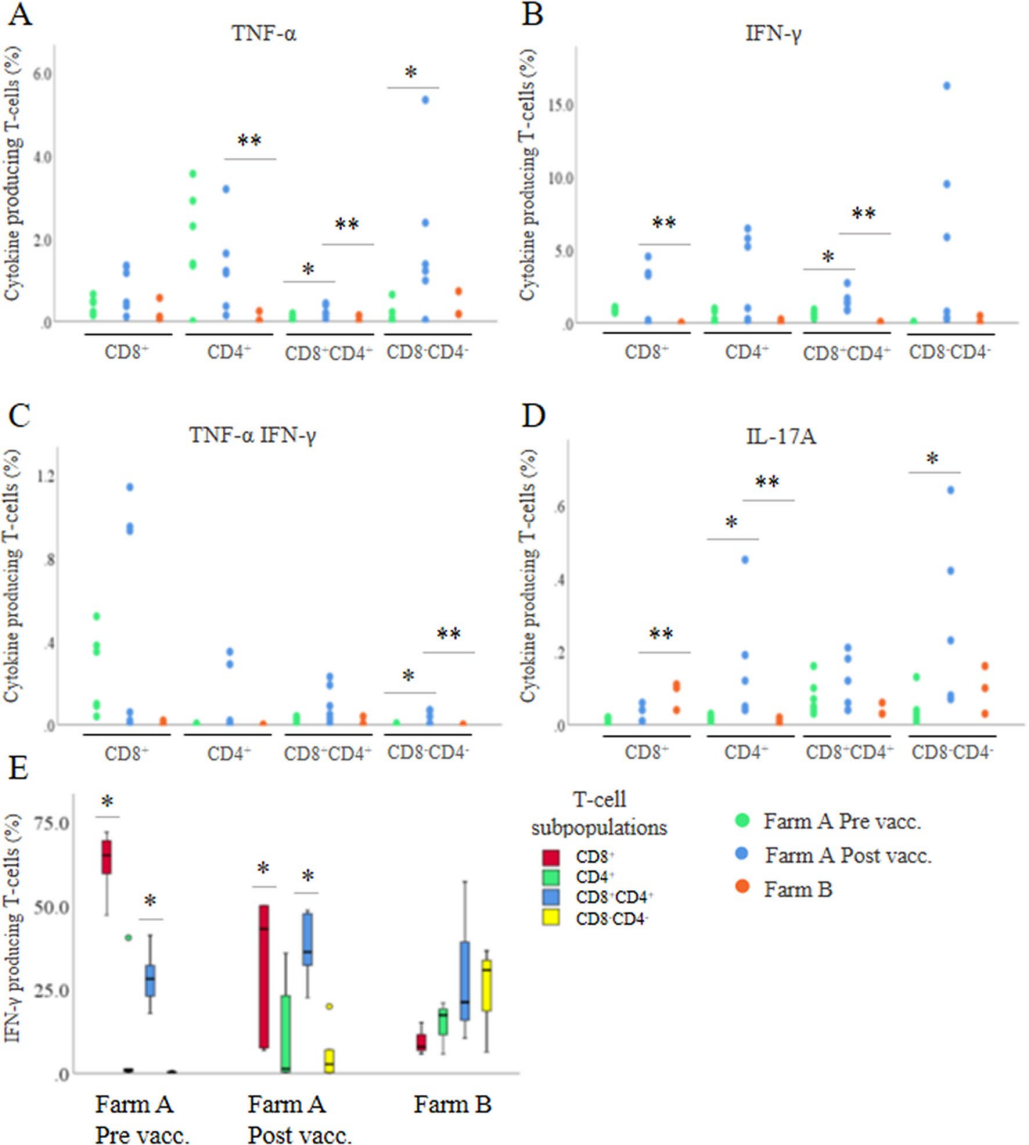
**Circulating*Mycoplasma hyopneumoniae*-specific cytokine producing T-cell subsets and IFN-γ production in blood of sows.** **A**–**D** Circulating *Mycoplasma hyopneumoniae-*specific cytokine producing T-cell subsets in blood of sows; **E** average IFN-γ production by the different T-cell subsets. PBMCs were stimulated with *M. hyopneumoniae* J strain bacterin and T-cell phenotype and cytokine production were assessed by flow cytometry. Farm A: endemically infected with *M. hyopneumoniae*; Farm B: free of *M. hyopneumoniae*. On farm A, sows (*n* = 6) were vaccinated against *M. hyopneumoniae* at 6 and 3 weeks before farrowing, blood samples were taken before the first vaccination (pre vacc.) and at the time of farrowing (post vacc.), on farm B blood was sampled of the sows (*n* = 3) at the time of farrowing. *, *P* < 0.05 between pre- and post-vaccination on farm A; **, *P* < 0.05 between farm A and B.

The CMI response 6 weeks after vaccination was further investigated by analyzing the presence of TNF-α, IFN-γ and IL-17A producing T-cells in the blood of sows after in vitro stimulation with *M. hyopneumoniae* bacterin. Six weeks after vaccination, *M. hyopneumoniae*-specific T-cells were present. CD3^+^ T-cells isolated from blood before vaccination did not proliferate upon in vitro simulation, while proliferation was observed for all sows after vaccination (Additional file 4). In blood of sows, vaccination resulted in a significant increase in IL-17A^+^ CD4^+^ T-cells (*P* = 0.03), TNF-α^+^ (*P* = 0.047) and IFN-γ^+^ (*P* = 0.04) CD8^+^CD4^+^ T-cells, and in TNF-α^+^ (*P* = 0.03), TNF-α^+^IFN-γ^+^ (*P* = 0.03) and IL-17A^+^ (*P* = 0.03) CD8^−^CD4^−^ T-cells compared to before vaccination (Figures 4A–D). At the time of farrowing, sows on farm B had significantly less IFN-γ^+^ (*P* = 0.02) CD8^+^ T-cells, TNF-α^+^ (*P* = 0.04) and IL-17A^+^ (*P* = 0.02) CD4^+^ T-cells, TNF-α^+^ (*P* = 0.04) and IFN-γ^+^ (*P* = 0.01) CD8^+^CD4^+^ T-cells, and TNF-α^+^IFN-γ^+^ (*P* = 0.02) CD8^−^CD4^−^ T-cells in their blood compared to sows on farm A at the time of farrowing (Figures 4A–D). However, IL-17A producing CD8^+^ T-cells were significantly more present in the blood of sows on farm B compared to sows on farm A at the time of farrowing (*P* = 0.02). Before vaccination, IFN-γ producing T-cells were mainly CD8^+^, while after vaccination there was a significant decrease in INF-γ^+^CD8^+^ T-cells (*P* = 0.03) and a significant increase in INF-γ^+^CD8^+^CD4^+^ T-cells (*P* = 0.02) (Figure 4E). No significant differences were observed in INF-γ producing T-cells in blood of sows between both farms at the time of farrowing (Figure 4E).

**Figure 5:**
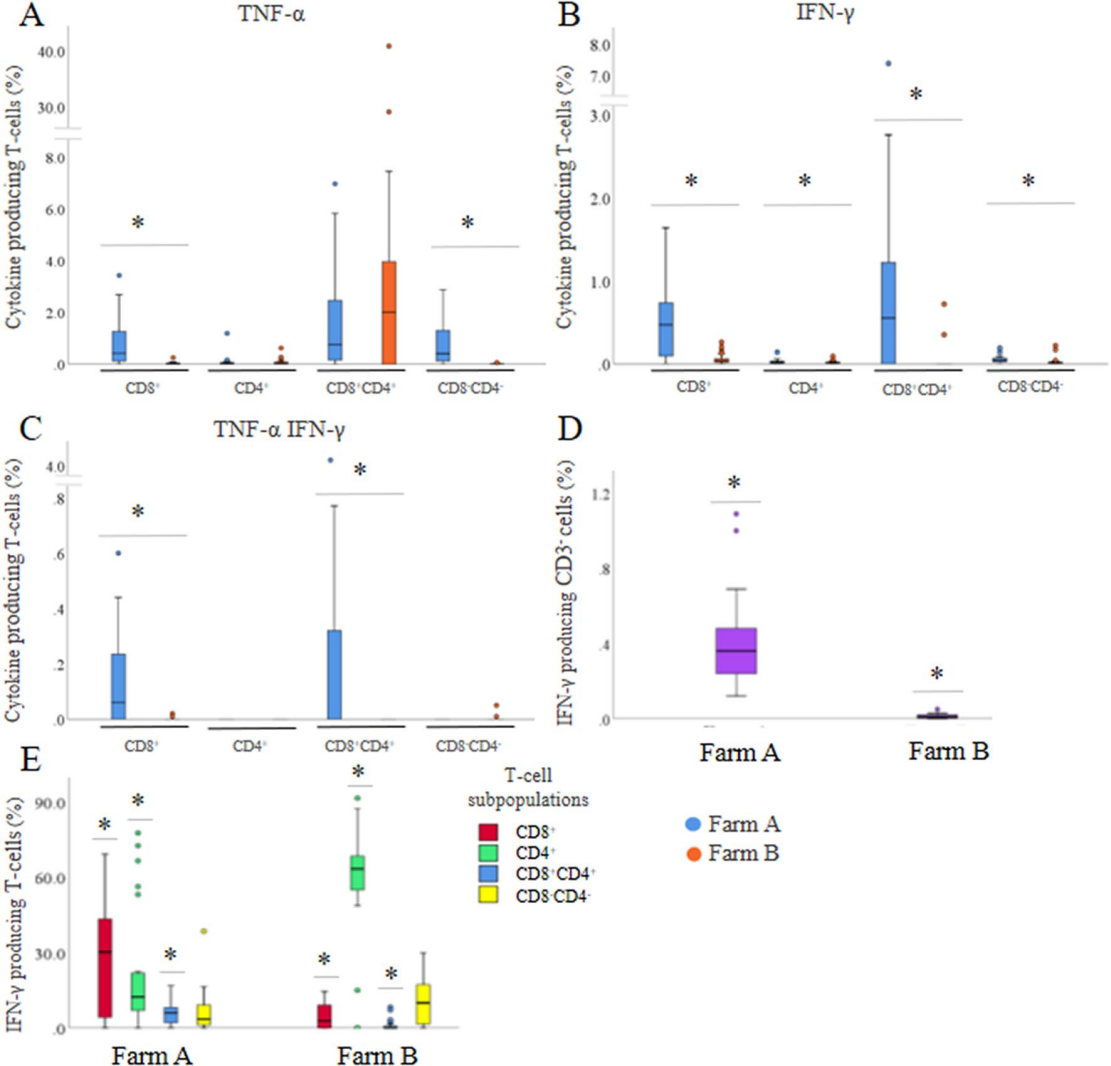
**Circulating** ***Mycoplasma hyopneumoniae*-specific cytokine producing T-cell subsets and IFN-γ production in blood of neonatal piglets.** **A**–**C** Circulating *Mycoplasma hyopneumoniae-*specific cytokine producing T-cell subsets in neonatal piglets; **D** IFN-γ production by CD3^−^ cells; **E** IFN-γ production in different T-cell subsets. PBMCs were stimulated with *M. hyopneumoniae* J strain bacterin and T-cell phenotype and cytokine production were assessed by flow cytometry. Farm A: *n* = 23 piglets, endemically infected with *M. hyopneumoniae*; Farm B: *n* = 24 piglets, free of *M. hyopneumoniae*. On farm A, sows were vaccinated against *M. hyopneumoniae* at 6 and 3 weeks before farrowing, while sows on farm B were not vaccinated. *, *P* < 0.05 between farm A and B.

To assess whether vaccine-induced T-cell responses are transferred from sow to piglets, the presence of TNF-α, IFN-γ and IL-17A producing T-cells was investigated in the blood of 2-day-old piglets after in vitro stimulation with *M. hyopneumoniae* bacterin. *M. hyopneumoniae*-specific cytokine producing T-cell subsets could be detected in the blood of the offspring. As shown in Figures 5A–C, piglets from vaccinated sows had significantly more TNF-α^+^ (*P* < 0.01), IFN-γ^+^ (*P* < 0.01) and TNF-α^+^IFN-γ^+^ (*P* < 0.01) CD8^+^ T-cells, INF-γ^+^ (*P* = 0.02) CD4^+^ T-cells, IFN-γ^+^ (*P* < 0.01) and TNF-α^+^IFN-γ^+^ (*P* < 0.01) CD8^+^CD4^+^ T-cells, and more TNF-α^+^ (*P* < 0.01) and IFN-γ^+^ (*P* < 0.01) CD8^−^CD4^−^ T-cells in their blood as compared to piglets from non-vaccinated, *M. hyopneumoniae* negative sows. In blood of piglets from vaccinated sows, significantly more IFN-γ^+^CD8^+^ (*P* < 0.01) and IFN-γ^+^CD8^+^CD4^+^ (*P* < 0.01) T-cells were present, while in blood of piglets from *M. hyopneumoniae* negative sows, significantly more IFN-γ^+^CD4^+^ (*P* < 0.01) T-cells were present (Figure 5E). Also, in blood of piglets from vaccinated sows, significantly more IFN-γ^+^CD3^−^ (*P* < 0.01) cells were present than in blood of piglets from *M. hyopneumoniae* negative sows (Figure 5D). These IFN-γ^+^CD3^−^ lymphocytes, in blood of piglets from *M. hyopneumoniae* vaccinated sows, were mainly CD3^−^CD8^−^ (89.1%).

### Effect of cross-fostering on transfer of humoral and CMI to the piglets

**Figure 6:**
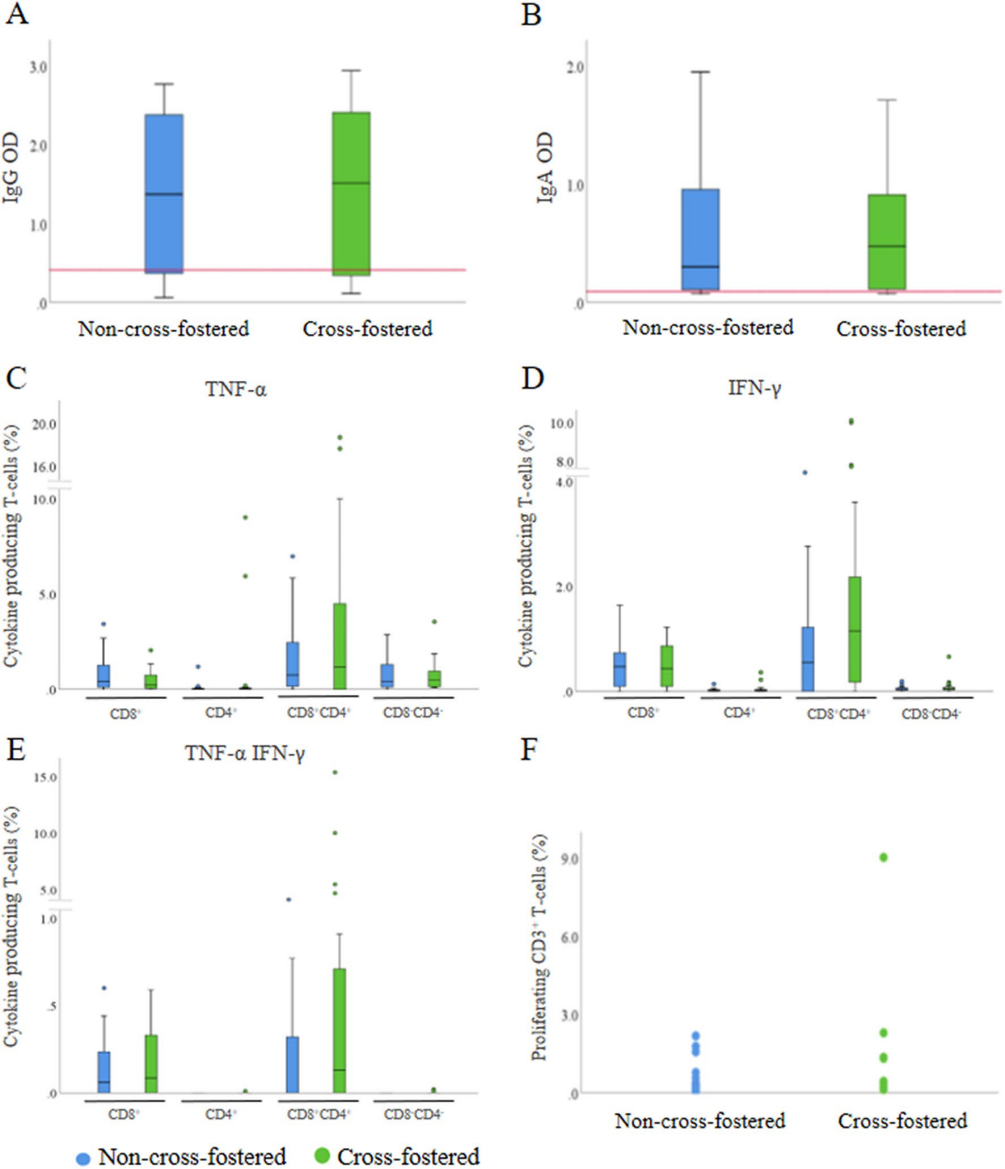
**Effect of cross-fostering on maternally-derived immunity in piglets.** **A**, **B** *Mycoplasma hyopneumoniae-*specific IgG and IgA levels; **C**–**E** percentage of *Mycoplasma hyopneumoniae-*specific cytokine producing T-cells; **F** *Mycoplasma hyopneumoniae-*specific proliferation of CD3^+^ T-cells in blood of 2-day-old non-cross-fostered (*n* = 23) and cross-fostered (*n* = 24) piglets on an endemically infected *M. hyopneumoniae* farm. Red line: cut-off optical density value for positive samples.

Cross-fostering did not influence the levels of *M. hyopneumoniae*-specific IgG and IgA antibodies in serum of piglets at 2-days of age as compared to non-cross-fostered piglets (Figures 6A and B).

To investigate if cross-fostering affected the uptake of CMI by the piglets, the presence of peripheral cytokine producing T-cells was compared between cross-fostered and non-cross-fostered 2-day-old piglets.

No significant differences were observed in the presence of cytokine producing T-cell populations in blood of 2-day-old non-cross-fostered and cross-fostered piglets after in vitro stimulation with *M. hyopneumoniae* bacterin (Figures 6C–E). In addition, no significant difference was observed in the percentage of proliferating CD3^+^ T-cells between non-cross-fostered and cross-fostered piglets upon *M. hyopneumoniae* stimulation (Figure 6F).

The potential correlation between the presence of cytokine producing T-cells in the blood of sows and their offspring was examined. Significant (*P* < 0.01) and positive correlations were found for TNF-α^+^ and TNF-α^+^IFN-γ^+^ CD8^+^ T-cells between non-cross-fostered and cross-fostered piglets and their birth sow and between cross-fostered piglets and their adoption sow (Additional file 5). The correlations ranged between 0.58 and 0.80 and were similar between piglets that had suckled their birth sow or a foster sow. Other correlations were very low and/or not significant.

## Discussion

In this study, we showed that *M. hyopneumoniae* vaccination of sows resulted in activation of *M. hyopneumoniae*-specific cytokine producing T-cells. These *M. hyopneumoniae-*specific T-cells were transferred to the offspring via colostrum and after isolation from the blood of the piglets, they were able to react with cytokine production upon in vitro stimulation with *M. hyopneumoniae* J strain bacterin. On the *M. hyopneumoniae* infected farm A, a non-vaccinated sow group was not included as the vaccinated sows served as their own control. The *M. hyopneumoniae-*specific immune status of the sows was compared before and after *M. hyopneumoniae* vaccination. As farm A was endemically infected, infection and possible triggering of the immune system of the sows during the study could not be excluded. Therefore, sows and piglets from a *M. hyopneumoniae* negative farm were included as control. On farm A the laryngeal swabs taken from the sows were all negative. The likelihood of detecting an infection with *M. hyopneumoniae* might have been higher when a tracheobronchial swab was used [32, 33]. It should be noted that both farms were different in terms of genetics, and some environmental conditions, and that these factors might have influenced the results of the present study in some way. As only one commercial *M. hyopneumoniae* vaccine was used, it is unsure whether the vaccine-induced responses observed in this study are also applicable to responses with other commercial vaccines. The adjuvant of the vaccine used in this study is a carbopol adjuvant. Studies done in mice demonstrated that carbopol has an impact on the adaptive immune response, directing it towards a Th1 response [34, 35].

To confirm that the transfer of *M. hyopneumoniae*-specific MDI from sows to piglets did occur, antibody levels in serum of sows before and after vaccination, in colostrum and in serum of 2-day-old piglets were investigated. On farm A, seroconversion was evident in four sows, but one sow remained negative for both *M. hyopneumoniae*-specific IgG and IgA antibodies, while another sow did not show an IgA response after vaccination. These results corroborated with previous studies indicating that seroconversion of sows upon *M. hyopneumoniae* vaccination can be variable [5, 16, 36]. As expected, the *M. hyopneumoniae*-specific IgG and IgA levels in colostrum corresponded with the sow’s serum levels at farrowing and the serum levels of 2-day-old piglets corresponded with the concentrations in serum and colostrum of their mother sow [15, 37–39]. The two non-responder sows were not excluded from the study since their CD3^+^ T-cells, isolated from blood after vaccination, showed a proliferation response to in vitro *M. hyopneumoniae* stimulation, indicating a positive CMI response upon *M. hyopneumoniae* vaccination [20]. The observed *M. hyopneumoniae*-specific immune response in sows following vaccination, might be a primary response or a booster of the existing *M. hyopneumoniae* immunity. Although all sows tested negative on nPCR performed on the laryngeal swabs, the farm was endemically infected with *M. hyopneumoniae.* Therefore, sows might have experienced a previous *M. hyopneumoniae* infection. On farm B, the *M. hyopneumoniae*-specific IgG and IgA levels in serum and colostrum of sows and serum of the 2-day-old piglets were low. At the time of farrowing, one sow had IgA levels in serum above the (in-house set) threshold value for positive samples, and two sows had IgG levels in colostrum above the threshold, which can be due to cross-reactivity between antibodies against *M. hyopneumoniae*, *M. hyosynoviae* and/or *M. flocculare* [40]. The serum of all sows was negative based on the commercial ELISA results.

Previous studies have shown that piglets from *M. hyopneumoniae* vaccinated sows are less often colonized with *M. hyopneumoniae* at weaning, although serum antibody levels are not correlated with the degree of protection [6]. Several authors suggested that CMI might play a role in the protection against *M. hyopneumoniae* infections [18–21]. As *M. hyopneumoniae* is primarily an extracellular pathogen, CD4^+^ T-cells, mainly the Th1 cells, are critical to protect against disease, probably due to IFN-γ activation of macrophages [25]. Stimulation of CD4^+^ T-cells, in pigs and a few other animal species such as chickens, mice and dogs, results in maturation to CD8^+^CD4^+^ T-cells, a T-cell subset with an antigen-specific memory function [41–44]. Six weeks after the first and three weeks after the second *M. hyopneumoniae* vaccination, there was a significant decrease in CD8^+^ T-cells in blood of sows. Vaccination increased the number of CD8^+^CD4^+^ T-cells, although the difference was not significant. This increase could be due to maturation of CD4^+^ T-cells. Furthermore, no significant difference was seen in the percentage of T-cell subpopulations in blood of sows at the time of farrowing on farm A versus farm B. These findings are in line with other studies, in which no significant differences were found in the percentage of T-cells between vaccinated and non-vaccinated groups [18, 19]. Unlike the results in blood, CD4^+^ T-cells were the dominant population in colostrum of sows on farm A, while on farm B the CD4^+^ T-cells were the least present. Previous studies demonstrated that CD8^+^ T-cells predominate over CD4^+^ T-cells in colostrum [8, 9, 39, 45], but these studies did not restimulate the isolated cells with *M. hyopneumoniae* antigen. The percentage of T-cell subsets in blood of 2-day-old piglets corresponds with the findings of previous research, investigating the T-cell subsets in blood of 1-day-old piglets [46]. CD4^+^ T-cells dominate over CD8^+^ T-cells and CD8^+^CD4^+^ T-cells are almost absent in newborn piglets [46, 47]. The percentage of CD8^+^CD4^+^ T-cells was significantly higher in the blood of 2-day-old piglets born from *M. hyopneumoniae* vaccinated sows compared to piglets from farm B. This could be explained by the fact that the vaccinated sows transferred *M. hyopneumoniae*-specific CD8^+^CD4^+^ memory T-cells via colostrum to the piglets, while the sows on farm B did not have *M. hyopneumoniae*-specific memory cells to transfer. However, comparing T-cell subsets with other studies remains difficult as PBMCs and CMCs in this study were first in vitro stimulated with *M. hyopneumoniae* bacterin before measuring the percentage of T-cell subpopulations. Although cells are stimulated in vitro, the measured percentage of the T-cell subsets is the total amount of cells present after *M. hyopneumoniae* stimulation and not only the *M. hyopneumoniae*-specific T-cells.

To the authors’ knowledge, no other studies have been conducted in which the effect of sow vaccination against *M. hyopneumoniae* on the presence of cytokine producing T-cells in the blood of sows was investigated. Vaccination of piglets against *M. hyopneumoniae* increased the number of IFN-γ secreting lymphocytes in the blood [18, 20, 21, 48]. Production of IFN-γ is crucial in the immune response. It directly promotes CMI as it stimulates a Th1 response, resulting in activation of natural killer cells and macrophages [26]. Most studies investigated the total amount of IFN-γ producing PBMCs with ELISPOT while the current study looked at the IFN-γ production by the individual T-cell subsets. In our study, the IFN-γ production was only significantly increased in CD8^+^CD4^+^ T-cells after vaccination. *M. hyopneumoniae* vaccination of sows resulted in a shift from IFN-γ production by CD8^+^ T-cells to IFN-γ production by CD8^+^CD4^+^ T-cells.

Bandrick et al. [23, 39] demonstrated the transfer of functional *M. hyopneumoniae*-specific lymphocytes and natural killer cells from sow to offspring. The present study further characterized the cytokine production of these *M. hyopneumoniae*-specific T-cells. In blood of 2-day-old piglets from *M. hyopneumoniae* vaccinated sows, significantly higher percentages of IFN-γ producing T-cells were present compared to 2-day-old piglets from non-vaccinated sows. In blood of piglets from vaccinated sows, IFN-γ is mainly produced by CD8^+^ T-cells. Furthermore, piglets from *M. hyopneumoniae* vaccinated sows had higher percentages of TNF-α producing CD8^+^ and CD8^−^CD4^−^ T-cells and of TNF-α and IFN-γ producing CD8^+^ and CD8^+^CD4^+^ T-cells. Lymphocytes with a memory function, such as CD8^+^CD4^+^ T-cells, which produce cytokines that work synergistically, e.g. TNF-α and IFN-γ, are expected to be important in the protection against pathogens [49]. Production of IFN-γ by B-cells has been described in mice [50]. Also, CD3^−^CD8^−^ lymphocytes, which included B-cells, produce significantly more IFN-γ in blood of piglets from vaccinated sows. Interestingly, IFN-γ producing B-cells were recently described in the context of PRRSV vaccination [51]. Further research is needed to elucidate the role of IFN-γ producing B-cells in the protection of pigs against pathogens.

The results obtained in our study confirm the transfer of *M. hyopneumoniae*-specific CMI from sows to their offspring. In addition, we showed that 2-day-old piglets from vaccinated sows have more *M. hyopneumoniae* specific cytokine producing T-cells in their blood than piglets from non-vaccinated sows on a *M. hyopneumoniae* negative farm. This indicates that vaccination of sows against *M. hyopneumoniae* in late gestation might have a positive effect on the pathogen specific CMI in their 2-day-old offspring.

It remains to be determined if the presence of *M. hyopneumoniae*-specific CMI in blood of neonatal piglets plays a role in protection against *M. hyopneumoniae* infections and if the passively acquired immunity interferes with the development of vaccine-induced immunity. Piglets with different statuses of *M. hyopneumoniae*-specific MDI were challenged [52]. They observed that piglets receiving full *M. hyopneumoniae* MDI, both cells and antibodies, had less and slower *M. hyopneumoniae* shedding but more lung lesions than piglets with no *M. hyopneumoniae* MDI. A study demonstrated that piglets from *M. hyopneumoniae* vaccinated sows did not show increased IgG levels after vaccination [53]. This in contrast to other studies showing that *M. hyopneumoniae* MDI had no effect on the development of *M. hyopneumoniae*-specific vaccine induced responses [28, 29]. However, based on the results of this study, we cannot elaborate further as the piglets were not *M. hyopneumoniae* challenged nor vaccinated.

On the *M. hyopneumoniae* positive farm, piglets were cross-fostered before colostrum ingestion and they were moved to a sow of a different breed. Cross-fostering had no impact on the transfer of *M. hyopneumoniae*-specific IgG and IgA antibodies, as previously shown [13]. Surprisingly, we were also not able to observe an impact of cross-fostering on the presence of *M. hyopneumoniae*-specific T-cells in the blood of 2-day-old piglets, in contrast to others [13]. Furthermore, the present study was not able to show differences in cytokine producing T-cells between 2-day-old piglets cross-fostered from a *M. hyopneumoniae* vaccinated dam to another vaccinated dam before colostrum ingestion on the one hand, and non-cross-fostered piglets on the other hand. In both, cross-fostered and non-cross-fostered piglets, CD3^+^ T-cells had the capacity to proliferate upon in vitro *M. hyopneumoniae* stimulation. Other studies suggested that colostral cells are expected to pass the intestinal epithelium only when the piglet suckled colostrum from its own mother sow [11, 12]. Previous research demonstrated that piglets cross-fostered from their *M. hyopneumoniae* vaccinated birth gilt to another *M. hyopneumoniae* vaccinated gilt before colostrum ingestion had a smaller DTH reaction than piglets cross-fostered 12 h or 20 h after birth [13]. A DTH reaction is the result of tissue damage caused by the activation of CD4^+^ T-cells, CD8^+^ T-cells, macrophages and natural killer cells following recognition of an antigen [54]. In contrast to this in vivo CMI response, we conducted an in vitro recall experiment to investigate the influence of cross-fostering on *M. hyopneumoniae* specific CMI in 2-day-old piglets.

The distribution of the T-cell subsets in blood of sows, colostrum and in blood of piglets after colostrum ingestion did not show similarities [39], which is in line with our findings. We found a significant positive correlation between TNF-α and TNF-αIFN-γ producing CD8^+^ T-cells in blood of *M. hyopneumoniae* vaccinated sows at the time of farrowing and the blood of their 2-day-old piglets, irrespective of cross-fostering. However, no other relevant correlations for cytokine producing T-cells between blood of sows and blood of piglets could be found. Unfortunately, data from cytokine production by colostral cells are lacking in this study due to the low cell yields from the colostral samples.

In conclusion, the present study showed that vaccination of sows against *M. hyopneumoniae* during late gestation induces *M. hyopneumoniae*-specific antibodies and poly-functional T-cells in sows, which are transferred via colostrum to neonatal piglets. *M. hyopneumoniae*-specific cytokine producing T-cells are found in blood of piglets from *M. hyopneumoniae* vaccinated sows. Further studies are warranted to investigate the role of these transferred maternally-derived poly-functional T-cells for the immune system in young piglets and for protection of piglets against *M. hyopneumoniae* infection.

## Supplementary Information

**Additional file 1. Gating strategy to assess cytokine production by T-cells with CytExpert software.**

**Additional file 2. Gating strategy applied on the T-cell proliferation assay with CytExpert software.**

**Additional file 3. Optical density values for** ***M. hyopneumoniae*-specific antibodies in serum of sows and 2-day-old piglets.** Farm A: endemically infected with *M. hyopneumoniae*; Farm B: free of *M. hyopneumoniae*. Optical density (OD) investigated with a commercial blocking ELISA (IDEIA™ Mycoplasma hyopneumoniae EIA kit, Oxoid Limited, Hampshire, UK). On farm A, sows were vaccinated against *M. hyopneumoniae *at 6 and 3 weeks before farrowing and blood samples were taken before the first vaccination (pre vacc.) and at the time of farrowing (post vacc.), on farm B blood was sampled of the sows at the time of farrowing. On farm A 47 piglets were sampled (24 cross-fostered and 23 non-cross fostered) and on farm B 24 piglets were sampled. Samples were considered positive if the OD of the sample was lower than 50% of the average OD of the buffer control.

**Additional file 4.*Mycoplasma hyopneumoniae*-specific proliferation of CD3**^**+**^** T-cells in blood of sows.** Farm A: endemically infected with *M. hyopneumoniae*; Farm B: free of *M. hyopneumoniae*. On farm A, sows (*n* = 6) were vaccinated against *M. hyopneumoniae *at 6 and 3 weeks before farrowing, blood samples were taken before the first vaccination (pre vacc.) and at the time of farrowing (post vacc.), on farm B blood was sampled of the sows (*n* = 3) at the time of farrowing.

**Additional file 5. Correlations for cytokine producing T-cell subsets between sows and 2-day-old piglets. **Significant (*P* < 0.01) positive correlations for TNF-α and TNF-αIFN-γ producing CD8^+^ T-cells in blood of sows at the time of farrowing and blood of 2-day-old piglets on an endemically infected* M. hyopneumoniae* farm. Correlation between (A, B) non-cross-fostered piglets and their birth sow; (C, D) cross-fostered piglets and their adoption sow; (E, F) cross-fostered piglets and their birth sow. r = correlation coefficient.

## Abbreviations

CMCs: Colostral mononuclear cells
CMI: Cell mediated immunity
DTH-reaction: Delayed-type hypersensitivity reaction
IFN-γ: Interferon gamma
Ig: Immunoglobulin
IL-17A: Interleukin 17A
MDI: Maternally-derived immunity
*M. hyopneumoniae*: *Mycoplasma hyopneumoniae*
nPCR: Nested polymerase chain reaction
OD: Optical density
PBMCs: Peripheral blood mononuclear cells
SD: Standard deviation
TNF-α: Tumor necrosis factor alpha

## References

[1] Pieters M, Maes D, Zimmermann JJ, Karriker LA, Ramirez A, Schwartz KJ, Stevenson GW, Zhang J (2019). Mycoplasmosis. Diseases of swine, Edition 11.

[2] Rycroft A, Maes D, Sibila M, Pieters M (2020). The general characteristics and classification of porcine *Mycoplasma* species. Mycoplasmas in Swine.

[3] Calsamiglia M, Pijoan C (2000). Colonisation state and colostral immunity to *Mycoplasma hyopneumoniae* of different parity sows. Vet Rec.

[4] Pieters M, Cline GS, Payne BJ, Prado C, Ertl JR, Rendahl AK (2014). Intra-farm risk factors for *Mycoplasma hyopneumoniae* colonization at weaning age. Vet Microbiol.

[5] Sibila M, Bernal R, Torrents D, Riera P, Llopart D, Calsamiglia M, Segales J (2008). Effect of sow vaccination against *Mycoplamsa hyopneumoniae* on sow and piglets colonization and seroconversion, and pig lung lesions at slaughter. Vet Microbiol.

[6] Arsenakis I, Michiels A, Schagemann G, Gomez-Duran CO, Boyen F, Haesebrouck F, Maes DGD (2019). Effects of pre-farrowing sow vaccination against *Mycoplasma hyopneumoniae* on offspring colonisation and lung lesions. Vet Rec.

[7] Kim YB (1975). Developmental immunity in the piglet. Birth Defects Orig Artic Seri.

[8] Pomorska-Mol M, Markowska-Daniel I, Bednarek D (2010). Flow cytometric analysis of leukocytes in porcine mammary secretion. Bull Vet Inst Pulawy.

[9] Hlavova K, Stepanova H, Faldyna M (2014). The phenotype and activation status of T and NK cells in porcine colostrum suggest these are central/effector memory cells. Vet J.

[10] Nechvatalova K, Kudlackova H, Leva L, Babickova K, Faldyna M (2011). Transfer of humoral and cell-mediated immunity via colostrum in pigs. Vet Immunol Immunopathol.

[11] Tuboly S, Bernath S, Glavits R, Medveczky I (1988). Intestinal absorption of colostral lymphoid cells in newborn piglets. Vet Immunol Immunopathol.

[12] Williams P (1993). Immunomodulation effects in intestinal absorbed maternal colostral leukocytes by neonatal pigs. Can J Vet Res.

[13] Bandrick M, Pieters M, Pijoan C, Baidoo SK, Molitor TW (2011). Effect of cross-fostering on transfer of maternal immunity to *Mycoplasma hyopneumoniae* to piglets. Vet Rec.

[14] Nguyen TV, Yuan L, Azevedo MSP, Jeong K, Gonzalez A, Saif LJ (2007). Transfer of maternal cytokines to suckling piglets: in vivo and in vitro models with implications for immunomodulation of neonatal immunity. Vet Immunol Immunopathol.

[15] Rautiainen E, Wallgren P (2001). Aspects of the transmission of protection against *Mycoplasma hyopneumoniae* from sow to offspring. Vet Med.

[16] Ruiz AR, Utrera V, Pijoan C (2003). Effect of *Mycoplasma hyopneumoniae* sow vaccination on piglet colonization at weaning. J Swine Health Prod.

[17] Djordjevic SP, Eamens GJ, Romalis LF, Nicholls PJ, Taylor V, Chin J (1997). Serum and mucosal antibody responses and protection in pigs vaccinated against *Mycoplasma hyopneumoniae* with vaccines containing a denatured membrane antigen pool and adjuvant. Aust Vet J.

[18] Thacker EL, Thacker BJ, Kuhn M, Hawkins PA, Waters WR (2000). Evaluation of local and systemic immune responses induced by intramuscular injection of a *Mycoplasma hyopneumoniae* bacterin to pigs. Am J Vet Res.

[19] Marchioro SB, Maes D, Flahou B, Pasmans F, Del Pozo SR, Vranckx K, Melkebeek V, Cox E, Wuyts N, Haesebrouck F (2013). Local and systemic immune responses in pigs intramuscularly injected with an inactivated *Mycoplasma hyopneumoniae* vaccine. Vaccine.

[20] Seo HW, Han K, Oh Y, Park C, Choo EJ, Kim S, Lee B, Chae C (2013). Comparison of cell-mediated immunity induced by three commercial singe-dose *Mycoplasma hyopneumoniae* bacterins in pigs. J Vet Med Sci.

[21] Martelli P, Saleri R, Cavalli V, De Angelis E, Ferrari L, Benetti M, Ferrarini G, Merialdi G, Borghetti P (2014). Systemic and local immune response in pigs intradermally and intramuscularly injected with inactivated *Mycoplasma hyopneumoniae* vaccines. Vet Microbiol.

[22] Garza-Moreno L, Segales J, Pieters M, Romagosa A, Sibila M (2017). Survey on *Mycoplasma hyopneumoniae* gilt acclimation practice in Europe. Porc Health Manag.

[23] Bandrick M, Pieters M, Pijoan C, Molitor TW (2008). Passive transfer of maternal *Mycoplasma hyopneumoniae*-specific cellular immunity to piglets. Clin Vaccine Immunol.

[24] Matthijs AMF, Auray G, Jakob V, Garcia-Nicolas O, Braun RO, Keller I, Bruggman R, Devriendt B, Boyen F, Guzman CA, Michiels A, Haesebrouck F, Collin N, Barnier-Quer C, Maes D, Summerfield A (2019). Systems immunology characterization of novel vaccine formulations for *Mycoplasma hyopneumoniae* bacterins. Front Immunol.

[25] Dobbs NA, Odeh AN, Sun X, Simecka JW (2009). The multifaceted role of T cell-mediated immunity in pathogenesis and resistance to Mycoplasma respiratory disease. Curr Trends Immunol.

[26] Schroder K, Hertzog PJ, Ravasi T, Hume DA (2004). Interferon-γ: an overview of signals, mechanisms and functions. J Leukoc Biol.

[27] Pappu R, Ramirez-Carrozzi V, Sambandam A (2011). The interleukine-17 cytokine family: critical players in host defence and inflammatory diseases. Immunol.

[28] Martelli P, Terreni M, Guazzetti S, Cavirani S (2006). Antibody response to *Mycoplasma hyopneumoniae* infection in vaccinated pigs with or without maternal antibodies induced by sow vaccination. J Vet Med.

[29] Bandrick M, Theis K, Molitor TW (2014). Maternal immunity enhances *Mycoplasma hyopneumoniae* vaccination induced cell-mediated immune responses in piglets. BMC Vet Res.

[30] Stärk KDC, Nicolet J, Frey J (1997). Detection of *Mycoplasma hyopneumoniae* by air sampling with nested PCR assay. Appl Environ Microbiol.

[31] Matthijs AMF, Auray G, Boyen F, Schoos A, Michiels A, Obdulio GN, Barut GT, Barnier-Quer C, Jakob V, Collin N, Devriendt B, Summerfield A, Haesebrouck F, Maes D (2019). Efficacy of three innovative bacterin vaccines against experimental infection with *Mycoplasma hyopneumoniae*. Vet Res.

[32] Fablet C, Marois C, Kobisch M, Madec F, Rose N (2010). Estimation of the sensitivity of four sampling methods for *Mycoplasma hyopneumoniae* detection in live pigs using a Bayesian approach. Vet Microbiol.

[33] Sponheim A, Alvarez J, Fano E, Schmaling E, Dee S, Hanson D, Wetzell T, Pieters M (2020). Comparison of the sensitivity of laryngeal swabs and deep tracheal catheters for detection of *Mycoplasma hyopneumoniae* in experimentally and naturally infected pigs early and late after infection. Vet Microbiol.

[34] Krahias G, Simon A, Wegmann F, Kok W, Ho L, Stevens D, Skehel J, Heeney JL, Moghaddam AE, Sattentau QJ (2010). Potent adaptive immune responses induced against HIV-1 gp 140 and influenza virus HA by a polyanionic carbomer. Vaccine.

[35] Gartlan KH, Krashias G, Wegmann F, Hillson WR, Scherer EM, Greenberg PD, Eisenbarth SC, Moghaddam AE, Sattentau QJ (2016). Sterile inflammation induced by Carbopol elicits robust adaptive immune responses in the absence of pathogen-associated molecular patterns. Vaccine.

[36] Thacker EL, Thacker BJ, Boettcher TB, Jayappa H (1998). Comparison of antibody production, lymphocyte stimulation, and protection induced by four commercial *Mycoplasma hyopneumoniae* bacterins. J Swine Health Prod.

[37] Morris CR, Gardner IA, Hietala SK, Carpenter TE, Anderson RJ, Parker KM (1994). Persistence of passively acquired antibodies to *Mycoplasma hyopneumoniae* in a swine herd. Prev Vet Med.

[38] Wallgren P, Bölske G, Gustafsson S, Mattsson S, Fossum C (1998). Humoral immune response to *Mycoplasma hyopneumoniae* in sows and offspring following an outbreak of mycoplasmosis. Vet Microbiol.

[39] Bandrick M, Ariza-Nieto C, Baidoo SK, Molitor TW (2015). Colostral antibody-mediated and cell-mediated immunity contributes to innate and antigen-specific immunity in piglets. Dev Comp Immunol.

[40] Gomes Neto JC, Strait EL, Raymond M, Ramirez A, Minion FC (2014). Antibody responses of swine following infection with *Mycoplasma hyopneumoniae*, *M. hyorhinis*, *M. hyosynoviae* and *M. flocculare*. Vet Microbiol.

[41] Zuckerman FA, Husmann RJ (1996). Functional and phenotypic analysis of porcine peripheral blood CD4/CD8 double-positive T cells. Immunol.

[42] Saalmüller A, Werner T, Fachinger V (2002). T-helper cells from naïve to committed. Vet Immunol Immunopathol.

[43] Nascimbeni M, Shin E, Chiriboga L, Kleiner DE, Rehermann B (2004). Peripheral CD4(+)CD8(+) T cells are differentiated effector memory cells with antiviral functions. Blood.

[44] McGill JL, Wang Y, Ganta CK, Boorgula GDY, Ganta RR (2018). Antigen-specific CD4^+^CD8^+^ double-positive T cells are increased in the blood and spleen during *Ehrlichia chaffeensis* infection in the canine host. Front Immunol.

[45] Le Jan C (1994). A study by flow cytometry of lymphocytes in sow colostrum. Res Vet Sci.

[46] Stepanova H, Samankova P, Leva L, Sinkora J, Faldyna M (2007). Early postnatal development of the immune system in piglets: the redistribution of T lymphocyte subsets. Cell Immunol.

[47] Borghetti P, De Angelis E, Saleri R, Cavalli V, Cacchioli A, Corradi A, Mocchegiani E, Martelli P (2006). Peripheral T lymphocyte changes in neonatal piglets: relationship with growth hormone (GH), prolactin (PRL) and cortisol changes. Vet Immunol Immunopathol.

[48] Park C, Jeong J, Choi K, Chae C (2016). Efficacy of a new bivalent vaccine of porcine circovirus type 2 and *Mycoplasma hyopneumoniae* (Fostera^TM^ PCV MH) under experimental conditions. Vaccine.

[49] Koinig HC, Talker SC, Stadler M, Ladinig A, Graage R, Ritzmann M, Hennig-Pauka I, Gerner W, Saalmüller A (2015). PCV2 vaccination induces IFN-/TNF- co-producing T cells with a potential role in protection. Vet Res.

[50] Harris DP, Goodrich S, Gerth AJ, Peng SL, Lund FE (2005). Regulation of IFN-γ production by B effector 1 cells: essention roles for T-bet and the IFN-γ receptor. J Immunol.

[51] Kick AR, Wolfe ZC, Amaral AF, Cortes LM, Almond GW, Crisci E, Gauger PC, Pittman J, Käser T (2021). Maternal autogenous inactivated virus vaccination boosts immunity to PRRSV in piglets. Vaccine.

[52] Pieters M, Oliveira S, Bandrick M, Baidoo S, Pijoan C, Molitor T (2008) Passive immunity to *Mycoplasma hyopneumoniae*: transfer and protective role. Retrieved from the University of Minnesota Digital Conservancy. https://hdl.handle.net/11299/140112

[53] Hodgins DC, Shewen PE, Dewey CE (2004). Influence of age and maternal antibodies on antibody responses of neonatal piglets vaccinated against *Mycoplasma hyopneumoniae*. J Swine Health Prod.

[54] Mak TW, Saunders ME, Mak TW, Saunders ME (2006). Allergy and hypersensitivity. The immune response.

