# Supplementary figures and images for: Transfer of Mycoplasma hyopneumoniae-specific cell mediated immunity to neonatal piglets
Source: Vet Res. 2021 Jun 30;52:96. doi: 10.1186/s13567-021-00968-0 (PMC8247214; doi:10.1186/s13567-021-00968-0)

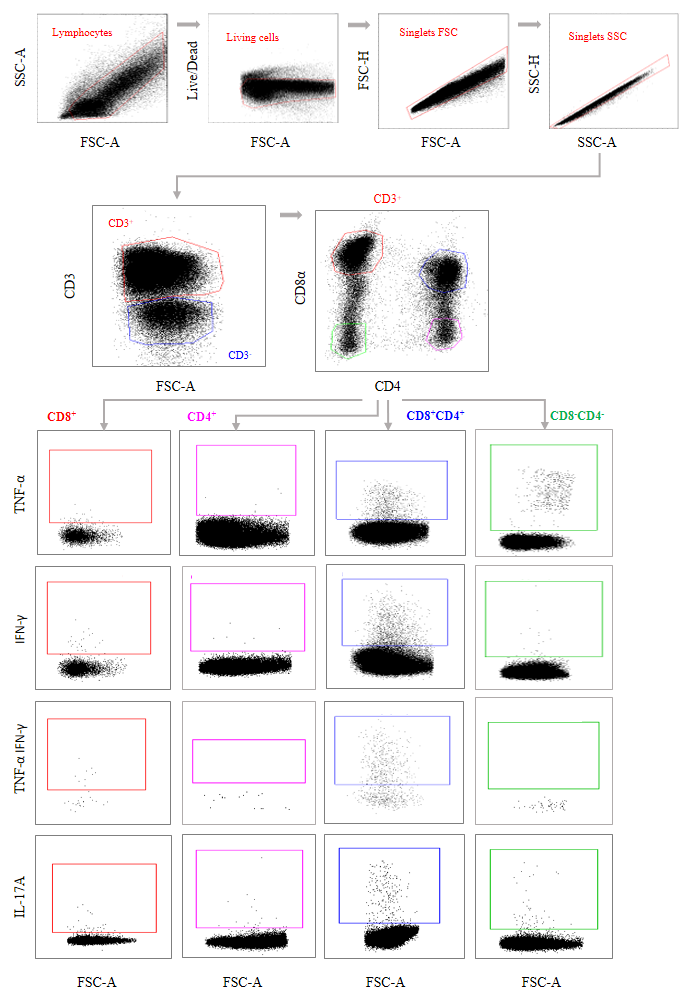

Supplement: Supplementary file 1 — Additional file 1. Gating strategy to assess cytokine production by T-cells with CytExpert software. [file 13567_2021_968_MOESM1_ESM.png]

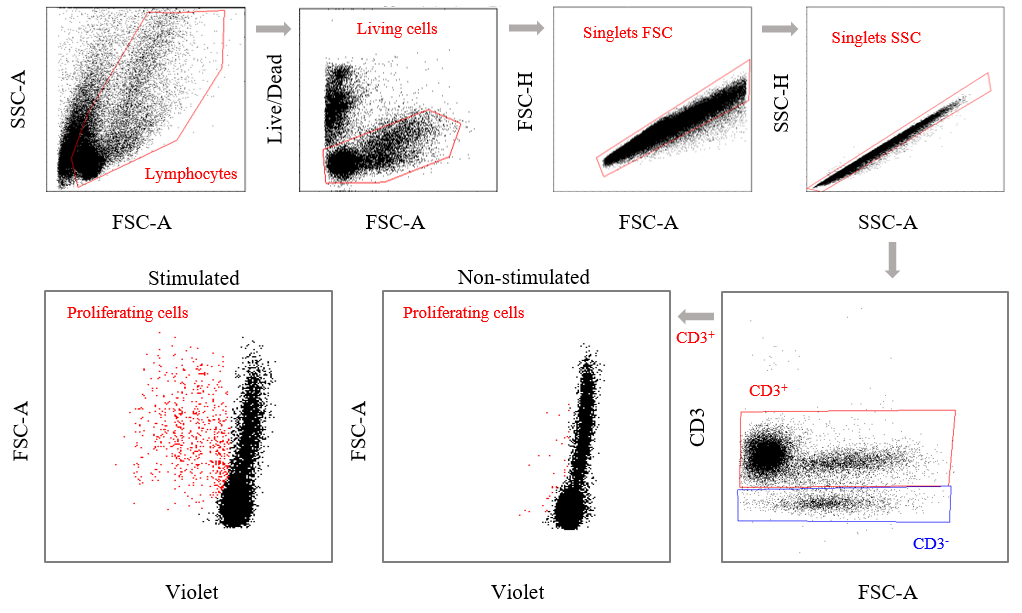

Supplement: Supplementary file 2 — Additional file 2. Gating strategy applied on the T-cell proliferation assay with CytExpert software. [file 13567_2021_968_MOESM2_ESM.png]

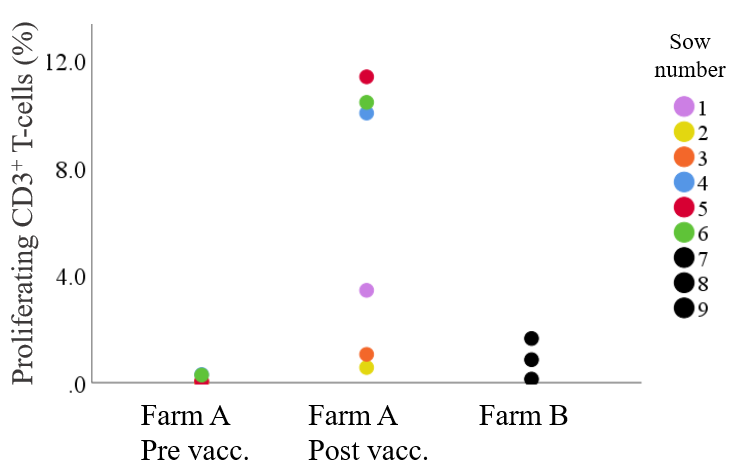

Supplement: Supplementary file 4 — Additional file 4.Mycoplasma hyopneumoniae-specific proliferation of CD3+ T-cells in blood of sows. Farm A: endemically infected with M. hyopneumoniae; Farm B: free of M. hyopneumoniae. On farm A, sows (n = 6) were vaccinated against M. hyopneumoniae at 6 and 3 weeks before farrowing, blood samples were taken before the first vaccination (pre vacc.) and at the time of farrowing (post vacc.), on farm B blood was sampled of the sows (n = 3) at the time of farrowing. [file 13567_2021_968_MOESM4_ESM.png]

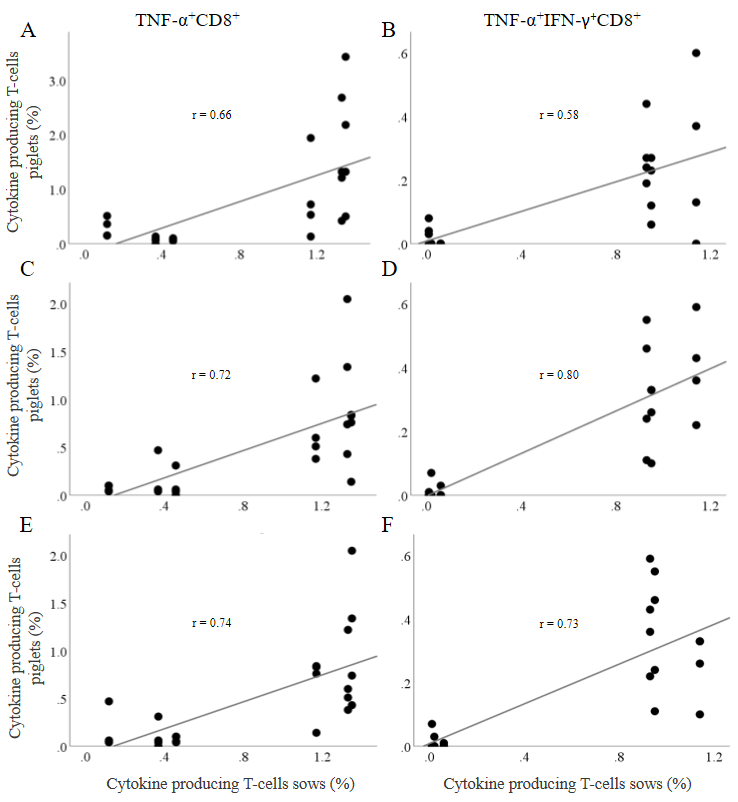

Supplement: Supplementary file 5 — Additional file 5. Correlations for cytokine producing T-cell subsets between sows and 2-day-old piglets. Significant (P < 0.01) positive correlations for TNF-α and TNF-αIFN-γ producing CD8+ T-cells in blood of sows at the time of farrowing and blood of 2-day-old piglets on an endemically infected M. hyopneumoniae farm. Correlation between (A, B) non-cross-fostered piglets and their birth sow; (C, D) cross-fostered piglets and their adoption sow; (E, F) cross-fostered piglets and their birth sow. r = correlation coefficient. [file 13567_2021_968_MOESM5_ESM.png]
