# Supplementary material for: Transfer of Mycoplasma hyopneumoniae-specific cell mediated immunity to neonatal piglets
Source: Vet Res. 2021 Jun 30;52:96. doi: 10.1186/s13567-021-00968-0 (PMC8247214; doi:10.1186/s13567-021-00968-0)
Supplement: Supplementary file 3 — Additional file 3. Optical density values for M. hyopneumoniae-specific antibodies in serum of sows and 2-day-old piglets. Farm A: endemically infected with M. hyopneumoniae; Farm B: free of M. hyopneumoniae. Optical density (OD) investigated with a commercial blocking ELISA (IDEIA™ Mycoplasma hyopneumoniae EIA kit, Oxoid Limited, Hampshire, UK). On farm A, sows were vaccinated against M. hyopneumoniae at 6 and 3 weeks before farrowing and blood samples were taken before the first vaccination (pre vacc.) and at the time of farrowing (post vacc.), on farm B blood was sampled of the sows at the time of farrowing. On farm A 47 piglets were sampled (24 cross-fostered and 23 non-cross fostered) and on farm B 24 piglets were sampled. Samples were considered positive if the OD of the sample was lower than 50% of the average OD of the buffer control. [file 13567_2021_968_MOESM3_ESM.docx]

| **Farm A** | | |  | **Farm B** | |
| --- | --- | --- | --- | --- | --- |
|  | Plate 1 | Plate 2 |  |  | Plate 3 |
|  | 50% buffer | 50% buffer |  |  | 50% buffer |
|  | 1.019 | 1.031 |  |  | 0.873 |
| Sows pre vacc. | 0.071 | 0.657 |  | Sows | 1.031 |
|  | 0.167 | 0.269 |  |  | 1.061 |
|  | 1.091 | 0.098 |  |  | 1.002 |
| Sows post vacc. | 0.022 | 0.025 |  | Piglets | 1.132 |
|  | 0.025 | 0.026 |  |  | 1.114 |
|  | 0.027 | 0.017 |  |  | 1.188 |
| Cross-fostered piglets | 0.026 | 0.019 |  |  | 1.191 |
|  | 0.021 | 0.029 |  |  | 1.185 |
|  | 0.024 | 0.03 |  |  | 1.119 |
|  | 0.024 | 0.018 |  |  | 0.936 |
|  | 0.025 | 0.022 |  |  | 0.882 |
|  | 0.024 | 0.026 |  |  | 1.231 |
|  | 0.023 | 0.024 |  |  | 1.196 |
|  | 0.033 | 0.025 |  |  | 1.353 |
|  | 0.025 | 0.034 |  |  | 1.189 |
|  | 0.024 | 0.037 |  |  | 1.246 |
|  | 0.021 | 0.031 |  |  | 1.031 |
|  | 0.022 | 0.039 |  |  | 1.135 |
| Non-cross-fostered piglets | 0.03 | 0.024 |  |  | 1.056 |
|  | 0.035 | 0.026 |  |  | 1.335 |
|  | 0.041 | 0.025 |  |  | 1.355 |
|  | 0.022 | 0.032 |  |  | 1.322 |
|  | 0.022 | 0.021 |  |  | 1.499 |
|  | 0.024 | 0.023 |  |  | 1.281 |
|  | 0.027 | 0.029 |  |  | 1.413 |
|  | 0.019 | 0.028 |  |  | 1.390 |
|  | 0.022 | 0.021 |  |  | 1.408 |
|  | 0.022 | 0.02 |  |  |  |
|  | 0.026 | 0.024 |  |  |  |
|  | 0.021 |  |  |  |  |
